# Association between social capital and mortality among community-dwelling older adults in Myanmar 2018–2022: a prospective cohort study

**DOI:** 10.1186/s44263-025-00137-x

**Published:** 2025-03-17

**Authors:** Yuka Ohaku, Yuki Shirakura, Yuiko Nagamine, Yuri Sasaki, Daisuke Takagi, Ikuma Nozaki, Than Win Nyunt, Reiko Saito, Yugo Shobugawa

**Affiliations:** 1 Division of International Health (Public Health), Graduate School of Medical and Dental Sciences, Niigata University, Niigata, Japan; 2 Department of Active Ageing, Graduate School of Medical and Dental Sciences, Niigata University, Niigata, Japan; 3 Department of Public Health, Institute of Science Tokyo, Tokyo, Japan; 4 Department of Public Health Policy, National Institute of Public Health, Saitama, Japan; 5 Department of Health and Social Behavior, School of Public Health, The University of Tokyo, Tokyo, Japan; 6 Bureau of International Health Cooperation, National Center for Global Health and Medicine, Tokyo, Japan; 7 Department of Geriatric Medicine, University of Medicine 1, Yangon, Yangon, Myanmar

**Keywords:** Healthy aging, Social capital, Social support, Mortality, Myanmar

## Abstract

**Background:**

Healthy aging is crucial in Asia given its rapidly aging society. Social capital, which refers to the resources derived from social networks, norms, and trust that facilitate cooperation and collective action within a community or society, has demonstrated health benefits for older adults. However, its impact varies by country. Most research focuses on high-income countries, with little attention on low- and middle-income countries.

**Methods:**

This prospective cohort study examined the effects of social capital on all-cause mortality among older adults in Myanmar from 2018 to 2022, using structured questionnaires based on the Japan Gerontological Evaluation Study. Multistage random sampling and face-to-face interviews were conducted with community-dwelling older adults aged 60 and above in Yangon and Bago in 2018. Subsequently, three waves of follow-up telephone surveys were conducted in 2020, 2021, and 2022. The questionnaires evaluated three components of social capital: civic participation, social cohesion, and social support, alongside baseline demographic information. Their impact on all-cause mortality was assessed using the Cox proportional hazards model with multiple imputations, adjusting for potential confounders including age, gender, body mass index, self-rated health, socioeconomic status, lifestyle, illness, and residential area.

**Results:**

A total of 1200 individuals were followed for an average of 2.6 years (3123 person-years), with 143 all-cause deaths observed among 1031 participants. Bivariate analyses showed that participants who died were more likely to be older, underweight, have shorter daily walking times, live in Bago, and have less social support. Higher social support was significantly associated with lower mortality after adjusting for all covariates (HR = 0.80, 95% CI 0.69–0.94). Specifically, instrumental support, defined as the exchange of practical assistance, such as receiving or providing care during illness, was found to be protectively associated with mortality. When stratified by residential area, significant associations were found only in Bago, a rural area. Among older adults in Myanmar, instrumental support was a more prominent protector against all-cause mortality than emotional support, especially in rural areas.

**Conclusions:**

Our findings indicate that social support networks play an important role in the survival of older adults in Myanmar, even under unstable social conditions.

**Supplementary Information:**

The online version contains supplementary material available at 10.1186/s44263-025-00137-x.

## Background

With the population aging globally, healthy aging has become a critical public health issue worldwide, as life expectancy continues to vary significantly across nations [1]. In 2019, Japan led with 84.3 years, contrasting sharply with several African nations below 50 years [2]. In Southeast Asia, disparities persist, from Myanmar at 69.1 years to the Maldives at 79.6 years. These variations highlight the importance of addressing both the biological and social factors influencing premature death.

In response to this trend, the United Nations established the Decade of Healthy Ageing (2021–2030) in 2020. This global collaboration aligns with the pursuit of the Sustainable Development Goals, aiming to improve the lives of older adults, their families, and their communities. It emphasizes the importance of identifying the factors affecting health and active aging worldwide, especially in low- and middle-income countries [3, 4].

As exemplified by the Social Determinants of Health concept, healthy aging is influenced not only by individual genetics and lifestyle habits but also by social factors, environmental conditions, and cultural backgrounds [5, 6]. These factors collectively contribute to global disparities in life expectancy with particular concern for low- and middle-income countries, including those in Southeast Asia, where the aging population is expected to grow rapidly over the next 20–30 years.

Among these factors, social capital has recently received significant attention as both a determinant and promoter of health [4] and longevity [7]. A growing body of literature indicates that social capital serves as a protective health factor among older adults, potentially reducing the risk of deterioration in psychological, physical, and cognitive health [8, 9]. Conceptually, social capital can be categorized into two dimensions: structural and cognitive [10]. Structural components encompass externally observable aspects of social organization, examining the degree and strength of social ties and social activities within society. When social capital is rich structurally, individuals may benefit from practical support and improved access to health-related information, encouraging healthier behaviors [11]. Cognitive components of social capital refer to individuals’ subjective perceptions of interpersonal trust, reciprocity, and willingness to share [10], which results in psychological security and tranquillity [12]. Social capital can be measured either collectively at the community level or individually. The effects of both individual- and community-level social capital on health varied across nations [8, 9, 13, 14]. Our study adopted the individual-level approach, reflecting how individuals perceive their social environment within community measures [15].

Social capital has a protective effect on health even in natural disasters and humanitarian crises, enhancing resilience and accelerating recovery and reconstruction. Kawachi et al. reported that older adults with abundant social capital before the Great East Japan earthquake had a lower risk of developing depression and post-traumatic stress symptoms [16–18]. Aldrich also reported that the readiness of individuals displaced by violence in Nigeria was significantly associated with abundant social capital. Similarly, in Uganda, the resilience of individuals facing food insecurity was influenced by the presence of social capital [19].

However, most social capital studies have been conducted in developed countries, with limited research available from low- and middle-income countries, including Southeast Asia [8, 20–22]. To the best of our knowledge, no study has shown an association between social capital and mortality among older adults in Southeast Asian countries. Therefore, the aim of this study was to investigate the effect of social capital on all-cause mortality among older adults in Myanmar.

## Methods

### Study design and study population

This longitudinal study used follow-up data from the “Healthy and Active Ageing in Myanmar (JAGES in Myanmar, commonly used abbreviation for the study, named after JAGES)” project, which adapts the Japan Gerontological Evaluation Study (JAGES) method, a nationwide survey aimed at exploring factors influencing healthy aging in Japan [23]. The study investigates the social determinants of healthy aging among community-dwelling older adults aged 60 years and above in two regions of Myanmar: Yangon and Bago. Our cohort, initiated in 2018, followed participants through three subsequent telephone surveys to track their survival status [24]. Yangon, the former capital with a population of 7.36 million, is a highly urbanized region and serves as the economic and commercial center of Myanmar [25]. By contrast, the Bago region, located north of Yangon with a population of 4.87 million, retains more rural characteristics and has a predominantly agricultural economy with fewer modern amenities and infrastructure developments. These two regions were selected to illustrate how urban and rural lifestyles affect the healthy aging of older adults in Myanmar.

**Fig. 1:**
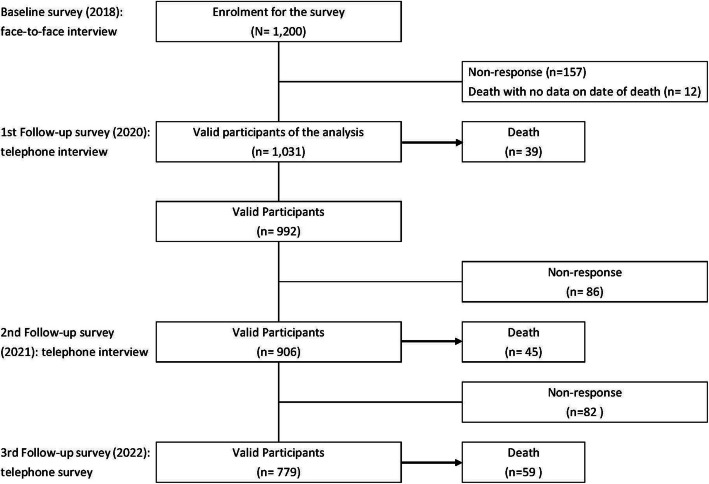
Flow chart of the Healthy and Active Ageing in Myanmar project, 2018–2022

A baseline survey was conducted from September to December in 2018, followed by three waves of follow-up telephone surveys in February 2020, March to April 2021, and May 2022 (Fig. 1). Data from the baseline and the three follow-up surveys were analyzed in this study.

A multistage random sampling method was applied in both Yangon and Bago at the baseline. First, six townships were randomly selected from each of the 45 townships in Yangon and 28 townships in Bago using population-proportionate sampling. Second, 10 wards were randomly selected from each township in Yangon, and 10 village tracts were selected from Bago. In Myanmar, a ward is the smallest unit of administrative division in urban areas, whereas a village tract is the corresponding level in rural areas. In cases where a village tract encompassed several villages, one village was randomly selected to represent the entire tract. Third, using the resident ledger list available from the township administrative office, eligible individuals were randomly selected to participate in the survey until the number of participants reached 10 for each ward or village tract. According to the WHO STEPS Surveillance Manual, which is a standardized system for collecting data on risk factors related to non-communicable diseases (NCDs) and other conditions, the ideal sample size was calculated to be 1200, with 600 sampled from Yangon and 600 from Bago [24, 26]. The sample size was calculated using the following standard formula.

$$n={Z}^{2}\frac{P(1-P)}{{e}^{2}}$$

*Z* represents the level of confidence, which was set at 1.96 in this study. *P* denotes the prevalence, which was assumed to be 0.3 at the start of the cohort study as an estimate for achieving healthy aging. *e* indicates the margin of error, set at 0.05. The calculated value of *n* was adjusted by incorporating a design effect of 1.5 to account for the response rate and multiplied by 2 to allow for stratified analysis between urban and rural populations. To ensure sufficient power, a margin was added, and the final sample size was determined to be 1200. Participants aged 60 years and above were included in the study if they resided in the selected ward or village tract and were not bedridden, did not have hearing impairments or severe dementia (as screened by the Abbreviated Mental Test [27, 28], and were not cohabitating with other participants.

A baseline survey was conducted in 2018 through face-to-face interviews. Trained surveyors and public health professionals visited participants’ homes and conducted interviews using a paper-based questionnaire. They visited the homes of 1083 older adults in Yangon, and 610 were at home at the time of the visit. Of these, 10 were excluded due to refusal to participate (*n* = 6), severe dementia, or being bedridden (*n* = 4) (Additional file 1: Fig. S1). In Bago, surveyors visited 1044 older adults, and 697 were at home at the time of the visit. Among them, 97 were excluded due to being from the same household (*n* = 51), having severe dementia, hearing problems, or being bedridden (*n* = 46). Consequently, a total of 1200 participants, 600 from Yangon and 600 from Bago, were enrolled. At the baseline, with the participant’s consent, we obtained their phone number and at least two additional phone numbers of family members or relevant persons. This was to facilitate future follow-up surveys in case the participant could not be contacted due to severe illness or death.

The first wave of follow-up telephone surveys was conducted in February 2020, followed by a second wave in March to April 2021, and a third wave in May 2022, following the same protocol. A shortened version of the questionnaire was developed based on the one used in the baseline survey. The questionnaires were designed to reflect the socioeconomic circumstances at the time of each follow-up survey, resulting in slight variations in the questions for each survey. Trained surveyors made phone calls to the participants. If they did not answer, a call was made to family members, relatives, community members, or the local municipal office to inquire about the participant’s status.

### Questionnaire

The structured questionnaire was developed based on the English version of the JAGES questionnaire. The content covered a broad range of individual physical and mental conditions, lifestyle status, and social aspects such as social capital and socioeconomic status. The JAGES questionnaire is not publicly available; however, researchers who wish to analyze and conduct studies using JAGES data can access it through the appropriate application and approval process [29]. The JAGES questionnaire was modified to fit the sociocultural context of Myanmar and translated into the local language, Burmese, via English. For example, religious gatherings, which are a common form of civic participation in Myanmar, were included as one of the response options. Additionally, adjustments were made to the Instrumental Activities of Daily Living (IADL) measurement items, such as excluding the question “Can you withdraw money from your own bank account?” since many older adults in Myanmar typically do not possess their own bank accounts. Consistency and accuracy were confirmed by translating it back into English. Questionnaire validation was conducted based on the Linguistic Validation Manual [30].

A pilot study was conducted at the Urban Health Center in Dagon Township, Yangon, to assess the validity and reliability of the study. Twenty-five adults aged 60 and above who visited the outpatient clinic participated, providing consent. The interviewers reviewed the order, flow, and clarity of the questionnaire, and it was revised and finalized based on their feedback. Although no major changes were made, minor adjustments were implemented, such as adding response options regarding modes of transportation and hobbies, as well as rephrasing questions that might cause confusion in answering.

### Measurements

#### All-cause mortality

Our outcome measure was all-cause mortality. The occurrence of death was ascertained during the phone interview with the respondent by asking, “How is [name of respondent at baseline survey]’s health status?” Possible responses were “excellent,” “good,” “fair,” “poor,” “dead,” or “I am not sure about their situation.” When the response indicated “dead,” we categorized the participants as deceased and inquired about the date of death. If this information could not be obtained, specific procedures were followed to assign an arbitrary date of death for survival analysis. For participants where only the year and month of death were obtained, the date of death was set at the middle of the month. In the first follow-up survey in 2020, participants with no data on the date of death were excluded from the analysis. In subsequent follow-up surveys, participants with no information on the date of death were treated as censored at the point when their survival was last confirmed.

#### Individual social capital

Our exposure measure was individual social capital. We assessed the three pillars of social capital adopted from a previous study [31]: civic participation, social cohesion, and social support. According to Harpham’s classification of social capital, social cohesion, and social support have a cognitive dimension, whereas civic participation has a structural dimension [4].

Civic participation was evaluated based on involvement in various activities, including religious groups, volunteer groups, sports groups/clubs, hobby groups, community meetings, or political meetings/events. We asked, “How often do you attend activities for the following groups?” The response options were “(1) four or more times a week,” “(2) two or three times a week,” “(3) once a week,” “(4) one to three times a month,” “(5) A few times a year,” or “(6) never.” We considered responses corresponding to options 1 through 4 as indicating participation and options 5 or 6 as no participation in civic activities.

Social cohesion comprised three variables: trust, norms of reciprocity, and attachment to the residential area. We asked, “Do you think people living in your area can be trusted in general?”, “Do you think people living in your area try to help others in most situations?”, and “Do you feel attached to the area you live in?” Responses for all questions were on a 5-point Likert scale: “very,” “moderately,” “neutral,” “not really,” or “not at all.” One point was assigned to a response of “very” or “moderately,” and the social cohesion score was calculated by summing the points for each question (score range: 0–3).

Social support was assessed by four items: receiving emotional support, providing emotional support, receiving instrumental support, and providing instrumental support. The corresponding questions were “Do you have someone who listens to your concerns and complaints?”, “Do you listen to someone’s concerns or complaints?”, “Do you have someone who looks after you when you are sick and confined to a bed for a few days?”, and “Do you look after someone when they are sick and confined to a bed for a few days?” All questions were multiple-choice with eight response options: “(1) spouse,” “(2) children living together,” “(3) children or relatives living apart,” “(4) brother/sister, relative, parents, grandchildren,” “(5) neighbor,” “6. friend,” “(7) other,” or “(8) I do not have such a person.” Responses corresponding to options 1 to 7 were considered indicative of either receiving or providing social support, and one point was assigned. The social support score was calculated by summing the points for each question (score range: 0–4).

#### Covariates

Covariates included age, gender, body mass index (BMI), self-rated health (SRH), socioeconomic status (SES), lifestyle behaviors, illness during the preceding year, and residential area. In this study, the term gender refers to the participant’s own self-reported perceived gender. Age was categorized into three groups (60–69, 70–79, or ≥ 80 years). BMI was calculated from height and weight objectively measured during the baseline survey and categorized into three groups (underweight, < 18.5 kg/m^2^; normal, 18.5 ≤ and < 25 kg/m^2^; overweight or obesity, ≥ 25 kg/m^2^). SRH was categorized into two groups (excellent/good or fair/poor). Indices of SES included educational level and wealth index. Educational level was classified into four categories (no school, Buddhist monastic school, some/full primary school, middle school or higher). The wealth index was categorized into three groups (low, middle, and high) based on a score calculated from the participant’s household asset items [32–34]. Lifestyle behaviors included smoking history (current smoker or ex/non-smoker), alcohol consumption (current drinker or ex/non-drinker), and walking time per day (< 30 min or ≥ 30 min). Illness during the preceding year was defined as the presence or absence of any self-reported illnesses within the past year (yes, no, or do not remember). The residential area was categorized into two groups (urban or rural). In this study, Yangon was defined as the urban area, whereas Bago was defined as the rural area.

The Katz Index, which was used for sensitivity analyses, measures basic activities of daily living (ADL) by assessing the ability to perform fundamental actions related to vision, hearing, walking, and memory. Participants were asked to rate their abilities on a four-point scale: no difficulty, some difficulty, great difficulty, and completely unable. The responses were scored.

### Statistical analysis

The characteristics of deceased and surviving participants were compared for all study variables using the chi-square test. Kaplan–Meier survival curves, along with the log-rank test, were fitted for each exposure (civic participation, social cohesion, and social support) to display the survival probabilities of the study cohort from the baseline survey in 2018 to the last telephone survey in 2022. Cox proportional hazards models were used to estimate hazard ratios (HRs) and 95% confidence intervals (95% CIs) for the three dimensions of social capital variables concerning all-cause mortality during the follow-up period.

The following multivariable models were constructed: Model 1 was adjusted for age and gender; Model 2 was additionally adjusted for SES; Model 3 was further adjusted for health-related factors such as BMI, SRH, illness during the preceding year, and lifestyle behaviors; and Model 4 was additionally adjusted for the residential area. The validity of the proportional hazard assumption was confirmed for all models by the Schoenfeld residual plots, justifying the use of Cox proportional hazard models.

We performed two additional analyses. First, for each component of civic participation, social cohesion, and social support, we calculated HRs using Cox proportional hazard models for all-cause mortality, adjusting for all covariates. For civic participation, HRs for participation in individual activity groups were calculated. For social cohesion, HRs for trust in neighbors, attachment to the residential area, and reciprocity were calculated. For social support, HRs for emotional and instrumental support receipt and provision were calculated separately. Second, we repeated the analyses, stratifying the data by the residential area.

As a sensitivity analysis, an additional examination was conducted. The total score of the Katz index, which serves as a fundamental indicator of basic ADL, was utilized as a proxy variable for diseases and severe disabilities. A Cox proportional hazards model, adjusted for all covariates, was employed to derive the HRs associated with all-cause mortality. The model assessed the effects of civic participation, social cohesion, and social support under both stratified and non-stratified conditions with respect to residential areas.

Regarding missing data, we performed multiple imputations using chained equations under a missing-at-random assumption. For the imputation, we used age, gender, SES, BMI, SRH, illness during the preceding year, lifestyle factors such as smoking, alcohol intake, walking time, and residential area. We created 20 imputed datasets and performed estimations. All statistical analyses were conducted using STATA17 (STATA Corp. LLC, College Station, TX, USA), and statistical significance was set at *P* < 0.05.

## Results

Of the 1200 participants at baseline, 169 were excluded at the first follow-up survey: 157 were lost to follow-up, and 12 were deceased with an unknown date of death. As a result, during 3123 person-years of follow-up over 4 years, with an average follow-up period of 2.6 years (standard deviation [SD] = 1.31), 143 all-cause deaths (men, *n* = 73; women, *n* = 70) were observed among 1031 valid participants (Fig. 1).

The mean age of the participants was 69.7 years (SD = 7.3), with 59.3% being women. The characteristics of the study cohort are presented in Table 1. Among the deceased participants, 31.5% were aged 80 and above compared to 9.0% among living participants. A higher proportion of deceased individuals were men (51.1%) compared to 39.1% of the living. Regarding weight, 40.6% of those who passed away were underweight compared to 28.4% of the survivors. Furthermore, 46.2% of the deceased participants exercised less than 30 min a day, in contrast to 33.9% of the living group. Geographically, 69.2% of the deceased were from Bago, compared to 56.1% of the living participants. Social support also differed, with 57.3% of the deceased both receiving and providing emotional and instrumental support, compared to 69.7% of among the survivors. Additionally, components of social support, such as receiving and providing emotional and instrumental support, were lower in the deceased group compared to the living. In summary, participants who died were more likely to be older, men, underweight, have shorter daily walking time, live in Bago, and have less involvement in social support.

**Table 1:** Participants’ characteristics

		**Overall**		**Non-death**		**Death**		P value*
		***N* = 1031**		***n* = 888**		***n* = 143**		
		***n***	**%**	***n***	**%**	***n***	**%**	
Age (years)	60–69	571	55.4	522	58.8	49	34.3	** < 0.001**
	70–79	335	32.5	286	32.2	49	34.3	
	≥ 80	125	12.1	80	9.0	45	31.5	
Gender	Male	420	40.7	347	39.1	73	51.1	**0.007**
	Female	611	59.3	541	60.9	70	49.0	
BMI (kg/m^2^)	< 18.5	310	30.1	252	28.4	58	40.6	**0.001**
	18.5–24.9	472	45.8	416	46.9	56	39.2	
	≥ 25	243	23.6	217	24.4	26	18.2	
	Missing	6	0.6	3	0.3	3	2.1	
SRH	Good	276	26.8	239	26.9	37	25.9	0.794
	Poor	755	73.2	649	73.1	106	74.1	
Education	No school	86	8.3	71	8.0	15	10.5	0.106
	Monastic school^1^	259	25.1	215	24.2	44	30.8	
	Some/full primary school^2^	362	35.1	312	35.1	50	35.0	
	Junior high school or higher	324	31.4	290	32.7	34	23.8	
Wealth index	Low	453	43.9	389	43.8	64	44.8	0.077
	Middle	379	36.8	317	35.7	62	43.4	
	High	197	19.1	180	20.3	17	11.9	
	Missing	2	0.2	2	0.2	0	0.0	
Alcohol consumption	Never/past drinker	979	95.0	846	95.3	133	93.0	0.251
	Current drinker	52	5.0	42	4.7	10	7.0	
Smoking history	Never/past smoker	780	75.7	673	75.8	107	74.8	0.803
	Current smoker	251	24.4	215	24.2	36	25.2	
Walking time	≥ 30 min/day	664	64.4	587	66.1	77	53.9	**0.004**
	< 30 min/day	367	35.6	301	33.9	66	46.2	
Illness during preceding year	No	508	49.3	441	49.7	67	46.9	0.527
	Yes	520	50.4	445	50.1	75	52.5	
	Do not remember	3	0.3	2	0.2	1	0.7	
Residential area	Yangon	434	42.1	390	43.9	44	30.8	**0.003**
	Bago	597	57.9	498	56.1	99	69.2	
Civic participation^3^	No	900	87.3	769	86.6	131	91.6	0.095
	Yes	131	12.7	119	13.4	12	8.4	
Religious groups	No	928	90.0	795	89.5	133	93.0	0.198
	Yes	103	10.0	93	10.5	10	7.0	
Volunteer groups	No	980	95.1	842	94.8	138	96.5	0.389
	Yes	51	5.0	46	5.2	5	3.5	
Sports groups/clubs	No	1024	99.3	882	99.3	142	99.3	0.975
	Yes	7	0.7	6	0.7	1	0.7	
Hobby groups	No	1024	99.3	882	99.3	142	99.3	0.975
	Yes	7	0.7	6	0.7	1	0.7	
Community meetings	No	1022	99.1	879	99.0	143	100.0	0.227
	Yes	9	0.9	9	1.0	0	0.0	
Political meetings/events	No	1029	99.8	887	99.9	142	99.3	0.139
	Yes	2	0.2	1	0.1	1	0.7	
Social cohesion^4^	0	15	1.5	15	1.7	0	0.0	0.388
	1	91	8.8	76	8.6	15	10.5	
	2	197	19.1	171	19.3	26	18.2	
	3	728	70.6	626	70.5	102	71.3	
Trust	No	264	25.6	225	25.3	39	27.3	0.623
	Yes	767	74.4	663	74.7	104	72.7	
Norms of reciprocity	No	117	11.4	103	11.6	14	9.8	0.527
	Yes	914	88.7	785	88.4	129	90.2	
Attachment	No	43	4.2	40	4.5	3	2.1	0.182
	Yes	988	95.8	848	95.5	140	97.9	
Social support^5^	0	11	1.1	4	0.5	7	4.9	** < 0.001**
	1	51	5.0	42	4.7	9	6.3	
	2	86	8.3	70	7.9	16	11.2	
	3	182	17.7	153	17.2	29	20.3	
	4	701	68.0	619	69.7	82	57.3	
Emotional support receipt	No	148	14.4	118	13.3	30	21.0	**0.015**
	Yes	883	85.7	770	86.7	113	79.0	
Emotional support provision	No	164	15.9	133	15.0	31	21.7	**0.042**
	Yes	867	84.1	755	85.0	112	78.3	
Instrumental support receipt	No	21	2.0	13	1.5	8	5.6	**0.001**
	Yes	1010	98.0	875	98.5	135	94.4	
Instrumental support provision	No	218	21.1	171	19.3	47	32.9	** < 0.001**
	Yes	813	78.9	717	80.7	96	67.1	

*BMI* body mass index, *SRH* self-rated health

*P value for chi-square test

^1^Monastic school refers to a school for reading and writing only

^2^Some/full primary school indicates both participants who stopped halfway through the program and those who completed the program

^3^ “Yes” indicates participation in any of the six kinds of groups, and “No” indicates never having participated in these activities

^4^Score calculated for each variable of social cohesion with responses of “very” or “moderately” scored as 1

^5^Score calculated for each variable of social support, with 1 point for “having experienced”

**Fig. 2:**
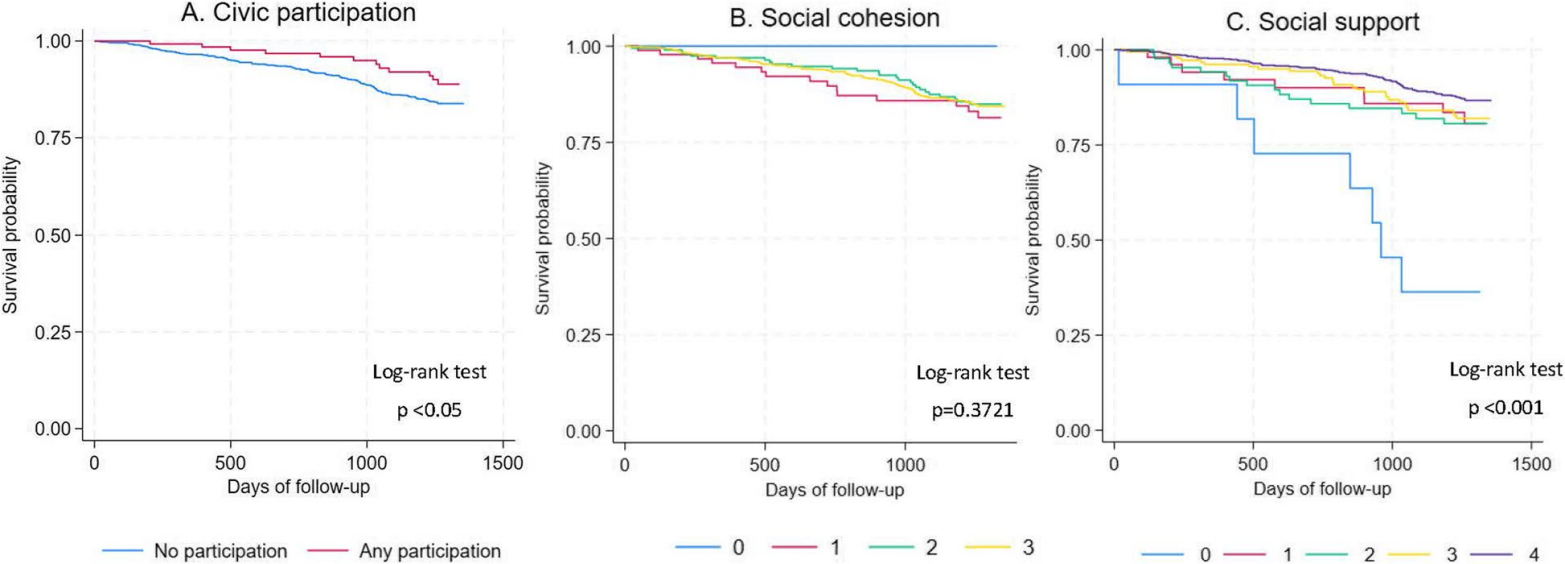
Kaplan–Meier survival estimates of social capital. Kaplan-Meier survival curves for civic participation (**A**), social cohesion (**B**), and social support (**C**). Panel B displays Kaplan-Meier survival curves by group, categorized based on the total score derived from summing each component of social cohesion. Panel C displays Kaplan-Meier survival curves by group, categorized based on the total score derived from summing each component of social support

Figure 2 presents the Kaplan–Meier survival plot of all-cause mortality stratified by the degree of (A) civic participation, (B) social cohesion, and (C) social support. We observed significantly shorter survival time for those lacking civic participation compared with those who had it (*p* < 0.05). Higher mortality risk was also observed in those with less social support (*p* < 0.001). No significant differences were shown between the curves stratified by social cohesion.

Tables 2, 3, and 4 present the results of the Cox proportional hazard modeling analysis, illustrating the association between each variable of individual social capital and all-cause mortality. No significant association was indicated between civic participation and mortality (Table 2) or between social cohesion and mortality (Table 3). By contrast, higher scores of social support consistently demonstrated a significant association with lower mortality, even after adjusting for all covariates (Model 4, HR = 0.80, 95% CI 0.69–0.94) (Table 4). There were 11 missing values for only three variables. The breakdown was as follows: 6 cases of BMI, 2 cases of wealth index, and 3 cases of illness during the preceding year. When analyzing the complete cases using raw data without multiple imputations, the results were similar. Given that the missing values were limited, the distribution of the variables was not affected, and therefore, the possibility of having distorted the results is considered to be minimal.

**Table 2:** Hazard ratios of civic participation for all-cause mortality

		Model 1			Model 2			Model 3			Model 4		
		HR	95%CI		HR	95%CI		HR	95%CI		HR	95%CI	
Civic participation	No	1.00			1.00			1.00			1.00		
	Yes	0.70	0.38	1.27	0.72	0.39	1.31	0.71	0.39	1.31	0.72	0.39	1.32
Age (years)	60–69	1.00			1.00			1.00			1.00		
	70–79	1.71**	1.15	2.55	1.78**	1.19	2.68	1.83**	1.21	2.79	1.83**	1.20	2.78
	≥ 80	5.07***	3.36	7.64	5.36***	3.48	8.26	5.14***	3.20	8.25	5.14***	3.20	8.24
Gender	Male	1.00			1.00			1.00			1.00		
	Female	0.56**	0.40	0.79	0.54**	0.38	0.77	0.55**	0.38	0.80	0.56**	0.38	0.81
Education	No school				1.00			1.00			1.00		
	Monastic school				0.90	0.49	1.67	0.92	0.49	1.71	0.92	0.49	1.70
	Some/full primary school				1.00	0.54	1.84	1.02	0.55	1.89	1.01	0.55	1.88
	Junior high school or higher				0.86	0.43	1.75	0.92	0.45	1.87	0.94	0.46	1.93
Wealth index	Low				1.00			1.00			1.00		
	Middle				1.52*	1.06	2.18	1.52*	1.05	2.20	1.59*	1.07	2.35
	High				0.73	0.40	1.32	0.67	0.35	1.26	0.73	0.36	1.47
BMI (kg/m^2^)	< 18.5							1.00			1.00		
	18.5–24.9							0.79	0.53	1.16	0.79	0.54	1.17
	≥ 25							1.02	0.61	1.71	1.04	0.62	1.77
SRH	Good							1.00			1.00		
	Poor							1.02	0.69	1.51	1.00	0.67	1.49
Illness during preceding year	Yes							1.00			1.00		
	No							1.06	0.75	1.48	1.06	0.75	1.49
Smoking history	Never/past (quit)							1.00			1.00		
	Everyday/sometimes							0.94	0.62	1.41	0.94	0.62	1.41
Alcohol consumption	Never/past (quit)							1.00			1.00		
	Current							2.01*	1.00	4.04	2.00	1.00	4.02
Walking time	≥ 30 min/day							1.00			1.00		
	< 30 min/day							1.35	0.96	1.90	1.36	0.96	1.91
Residential area	Yangon										1.00		
	Bago										1.15	0.71	1.86

Multiple imputation by chained equations was performed using age, gender, education, wealth index, BMI, SRH, illness during preceding year, smoking history, alcohol consumption, walking time, and residential area. Model 1 was adjusted for age and gender. Model 2 was adjusted for education and wealth index in addition to Model 1. Model 3 was adjusted for BMI, SRH, illness during preceding year, smoking history, alcohol consumption, and walking time in addition to Model 2. Model 4 was adjusted for residential area in addition to Model 3

*BMI* body mass index, *SRH* self-rated health, *HR* hazard ratio, *CI* confidence interval

^*^P < 0.05

**P < 0.01

***P < 0.001

**Table 3:** Hazard ratios of social cohesion for all-cause mortality

		Model 1			Model 2			Model 3			Model 4		
		HR	95%CI		HR	95%CI		HR	95%CI		HR	95%CI	
Social cohesion		1.05	0.83	1.33	1.06	0.83	1.34	1.06	0.84	1.35	1.05	0.83	1.34
Age (years)	60–69	1.00			1.00			1.00			1.00		
	70–79	1.76**	1.19	2.62	1.82**	1.22	2.73	1.88**	1.24	2.85	1.88**	1.24	2.85
	≥ 80	5.29***	3.52	7.94	5.53***	3.60	8.50	5.32***	3.32	8.52	5.31***	3.31	8.51
Gender	Male	1.00			1.00			1.00			1.00		
	Female	0.58**	0.42	0.81	0.56**	0.39	0.79	0.56**	0.39	0.82	0.57**	0.39	0.83
Education	No school				1.00			1.00			1.00		
	Monastic school				0.89	0.48	1.66	0.91	0.49	1.69	0.90	0.49	1.68
	Some/full primary school				0.98	0.53	1.80	1.00	0.54	1.86	1.00	0.54	1.85
	Junior high school or higher				0.83	0.41	1.68	0.88	0.43	1.80	0.91	0.44	1.86
Wealth index	Low				1.00			1.00			1.00		
	Middle				1.52*	1.06	2.18	1.53*	1.05	2.21	1.59*	1.07	2.35
	High				0.73	0.40	1.32	0.67	0.35	1.27	0.73	0.36	1.48
BMI (kg/m^2^)	< 18.5							1.00			1.00		
	18.5–24.9							0.78	0.53	1.15	0.79	0.54	1.17
	≥ 25							1.01	0.60	1.70	1.04	0.61	1.76
SRH	Good							1.00			1.00		
	Poor							1.02	0.69	1.52	1.00	0.67	1.50
Illness during preceding year	Yes							1.00			1.00		
	No							1.06	0.75	1.49	1.06	0.76	1.50
Smoking history	Never/past (quit)							1.00			1.00		
	Everyday/sometimes							0.94	0.62	1.41	0.94	0.62	1.41
Alcohol consumption	Never/past (quit)							1.00			1.00		
	Current							1.99	0.99	4.00	1.98	0.99	3.98
Walking time	≥ 30 min/day							1.00			1.00		
	< 30 min/day							1.35	0.96	1.90	1.36	0.97	1.92
Residential area	Yangon										1.00		
	Bago										1.15	0.71	1.87

Multiple imputation by chained equations was performed using age, gender, education, wealth index, BMI, SRH, illness during preceding year, smoking history, alcohol consumption, walking time, and residential area. Model 1 was adjusted for age and gender. Model 2 was adjusted for education and wealth index in addition to Model 1. Model 3 was adjusted for BMI, SRH, illness during preceding year, smoking history, alcohol consumption, and walking time in addition to Model 2. Model 4 was adjusted for residential area in addition to Model 3

*BMI* body mass index, *SRH* self-rated health, *HR* hazard ratio, *CI* confidence interval

^*^P < 0.05

**P < 0.01

***P < 0.001

**Table 4:** Hazard ratios of social support for all-cause mortality

		Model 1			Model 2			Model 3			Model 4		
		HR	95%CI		HR	95%CI		HR	95%CI		HR	95%CI	
Social support		0.83*	0.71	0.96	0.83*	0.71	0.97	0.80**	0.69	0.94	0.80**	0.69	0.94
Age (years)	60–69	1.00			1.00			1.00			1.00		
	70–79	1.72**	1.16	2.56	1.79**	1.19	2.68	1.81**	1.20	2.75	1.81**	1.19	2.74
	≥ 80	4.74***	3.12	7.21	4.99***	3.21	7.74	4.52***	2.78	7.34	4.51***	2.78	7.33
Gender	Male	1.00			1.00			1.00			1.00		
	Female	0.58**	0.41	0.80	0.56**	0.39	0.79	0.56**	0.39	0.81	0.57**	0.39	0.83
Education	No school				1.00			1.00			1.00		
	Monastic school				0.90	0.48	1.66	0.90	0.48	1.67	0.89	0.48	1.66
	Some/full primary school				0.98	0.53	1.81	1.00	0.54	1.86	0.99	0.54	1.84
	Junior high school or higher				0.88	0.43	1.79	0.94	0.46	1.93	0.97	0.47	2.00
Wealth index	Low				1.00			1.00			1.00		
	Middle				1.52*	1.06	2.18	1.53*	1.06	2.22	1.60*	1.08	2.36
	High				0.72	0.39	1.30	0.66	0.35	1.25	0.72	0.36	1.45
BMI (kg/m^2^)	< 18.5							1.00			1.00		
	18.5–24.9							0.75	0.51	1.11	0.76	0.52	1.13
	≥ 25							0.91	0.54	1.53	0.94	0.55	1.59
SRH	Good							1.00			1.00		
	Poor							1.02	0.68	1.51	1.00	0.67	1.49
Illness during preceding year	Yes							1.00			1.00		
	No							1.06	0.76	1.50	1.07	0.76	1.50
Smoking history	Never/past (quit)							1.00			1.00		
	Everyday/sometimes							0.95	0.63	1.43	0.95	0.63	1.43
Alcohol consumption	Never/past (quit)							1.00			1.00		
	Current							1.95	0.97	3.92	1.95	0.97	3.90
Walking time	≥ 30 min/day							1.00			1.00		
	< 30 min/day							1.46*	1.03	2.07	1.47*	1.04	2.09
Residential area	Yangon										1.00		
	Bago										1.15	0.71	1.85

Multiple imputation by chained equations was performed using age, gender, education, wealth index, BMI, SRH, illness during preceding year, smoking history, alcohol consumption, walking time, and residential area. Model 1 was adjusted for age and gender. Model 2 was adjusted for education and wealth index in addition to Model 1. Model 3 was adjusted for BMI, SRH, illness during preceding year, smoking history, alcohol consumption, and walking time in addition to Model 2. Model 4 was adjusted for residential area in addition to Model 3

*BMI* body mass index, *SRH* self-rated health, *HR* hazard ratio, *CI* confidence interval

^*^P < 0.05

**P < 0.01

***P < 0.001

Additional file 1: Fig. S2 shows the results of the Cox proportional hazard modeling analysis, demonstrating the effects of each component of civic participation, social cohesion, and social support on all-cause mortality, adjusting for all covariates. Instrumental support receipt and provision were protectively associated with mortality (HR = 0.31, 95% CI 0.15–0.64; HR = 0.61, 95% CI 0.42–0.90, respectively). Furthermore, the results stratified by residential area showed that social support and some components of social support, namely emotional support receipt and instrumental support receipt and provision, were protective against mortality in Bago (HR = 0.72, 95% CI 0.61–0.86; HR = 0.50, 95% CI 0.31–0.81; HR = 0.21, 95% CI 0.09–0.48; HR = 0.50, 95% CI 0.32–0.80, respectively) (Additional file 1: Fig. S3). However, this association was not observed in Yangon.

Additional file 2: Table. S1 illustrates the results of the modeling analysis that incorporates the Katz index, a fundamental indicator of basic ADL, into the full model. Among the three indicators of social capital, only social support was found to have a statistically significant association with a reduction in mortality (HR = 0.84, 95%CI 0.71–0.98), which was observed exclusively in the Bago region (HR = 0.78, 95%CI 0.64–0.94) when stratified by residential area. This association was not detected in Yangon (HR = 1.03, 95%CI 0.72–1.49).

## Discussion

To the best of our knowledge, this was the first prospective cohort investigation into the relationship between social capital and all-cause mortality among community-dwelling older adults in Myanmar. It found that higher levels of social support were associated with reduced all-cause mortality, whereas civic participation and social cohesion showed no significant effects. These results provide valuable public health insights aimed at promoting healthy aging among Myanmar’s older population.

Consistent with previous meta-analysis [35], our findings affirm that social support reduces mortality risk among older adults in Myanmar. This aligns with the Alameda County Study [36], which first identified lower mortality rates linked to social connections and support. We observed a protective effect of instrumental support exchanges (i.e., receiving and providing support), with emotional support showing a suggestive protective trend. Myanmar’s collectivist culture fosters strong family and community bonds, emphasizing mutual assistance [37]. With around 90% of Myanmar’s population adhering to Theravada Buddhism [38, 39], mutual assistance grounded in religious teachings is commonplace. In Myanmar, many Buddhists participate in weekend charity events at temples, offering alms or food to the needy [40]. This cultural context facilitates easier exchange of instrumental support among older adults, providing them with a protective social network [41]. Conversely, those lacking such support networks may face increased vulnerability to adverse health outcomes.

Stratifying by residential area revealed that the significant association between social support and mortality persisted only in Bago (rural area) and not in Yangon (urban area). This underscores the influence of urban and rural sociocultural contexts. Urban settings typically offer better service availability, accessibility, and infrastructure including healthcare, education, and transportation systems, reducing reliance on mutual assistance for daily needs [42, 43]. By contrast, economic disparities between urban and rural areas are often significant, with rural areas facing higher poverty rates and limited access to essential services and job opportunities. As a result, rural areas tend to nurture stronger familial and community ties, facilitating more substantial exchanges of physical support crucial for daily life [41, 44]. Consequently, in resource-limited rural settings, close-knit networks providing instrumental support may directly impact survival rates.

During our study’s follow-up, Myanmar experienced significant social, economic, public health, and political turmoil due to the COVID-19 pandemic since 2020, and subsequent political unrest from 2021. These turbulent events likely impacted the lives and health of older adults in Myanmar. Existing literature, such as a systematic review on the health impact of political instability and war on older adults in low- and middle-income countries [45] underscores the protective role of social support across various health outcomes, including quality of life [46], post-traumatic stress disorder [47], depression [48], and anxiety [49], as also observed in natural disasters [17, 50, 51]. It is important to note that our investigation only considered exposure factors at baseline in 2018 and did not sufficiently examine how the political instability and public health crisis resulting from the COVID-19 pandemic have affected the health of older adults in Myanmar. This aspect remains a significant challenge for future research.

Regarding the association between civic participation and mortality, our findings did not reach statistical significance. This contrasts with previous research indicating a positive impact of civic participation on mortality [20, 52]. Several explanations are plausible. First, while it is common for people in Myanmar to regularly visit nearby Buddhist pagodas, temples, and monasteries for prayer and socializing with friends, only 10.5% of participants reported participating in religious group activities. Similarly, participation rates in other social activities were generally low (0.7% for sports groups/clubs to 5.2% for volunteer groups). For older adults in Myanmar, these activities are part of daily life and are not considered participating in civic activities. This misclassification may explain why civic participation showed no association with mortality in our study. Given that the sociocultural concept of civic participation may vary by country, our questionnaire may not fully capture the sociocultural context of Myanmar. Second, one possible pathway through which social participation reduces mortality is by fostering social integration, thereby enhancing social networks and support [53]. However, if interpersonal ties and social connections are already robust within the cultural context of Myanmar, as discussed above, the additional benefit of participating in social groups may be minimal.

The evidence regarding the association between social cohesion and mortality has been inconsistent, with some studies indicating a positive effect [54–56], while others finding no significant association [20, 57]. In our study, social cohesion did not impact mortality. One possible explanation is that individual-level social cohesion does not always have a positive impact on health outcomes. As noted by Villalonga-Olives et al., social cohesion may have negative effects on health—the so-called “dark side” of social capital [58]. In a cohesive society, social networks can contribute to negative effects on health and may lead to the exclusion of non-members from accessing resources and opportunities [59]. This phenomenon may be particularly relevant in collectivist cultures such as those in Asia, where strong group ties and communal relationships are emphasized. In Myanmar, close family connections and community-based support systems are deeply embedded in society. These cultural characteristics may have influenced the lack of association between social cohesion and mortality observed in our study, suggesting that the impact of social cohesion on health may vary depending on the broader social and cultural context. Therefore, this issue warrants examination across a variety of social and cultural contexts. Moreover, studies have shown complex interactions between individual- and community-level social cohesion in influencing health outcomes. High levels of trust among individuals in communities with low overall trust levels may lead to poorer health outcomes. Conversely, individuals with lower trust levels in communities with higher overall trust levels may not necessarily experience better health outcomes [60, 61]. While our study focused solely on individual-level social cohesion, future research should explore community-level social cohesion for a more comprehensive understanding of its impact on health outcomes.

This study had several limitations. First, with 143 deaths among 1031 valid participants, the study may have had insufficient statistical power to detect certain associations. Of course, a larger sample size would not necessarily result in statistically significant results, but it would improve the accuracy of the estimates and lead to more accurate results. We hope that future follow-up studies will clarify this point. Second, 168 individuals (16% of the sample) were lost to follow-up, potentially introducing selection bias. Third, the determination of deaths relied on reports from telephone interviews, as the death registration system is weak in terms of coverage and completeness in Myanmar. While the reliability of death reports from respondents was high, this methodological limitation exists in data collection in Myanmar. Fourth, the average follow-up period of 2.6 years may have been insufficient to capture certain associations, as the effects of social capital on mortality may require more time to manifest. Future surveys with longer follow-up periods could address this issue. Moreover, the social capital used as the exposure variable in this study was measured only at baseline, and potential impacts of events such as the pandemic or political instability on social capital during the follow-up period were not accounted for. This limitation should be considered when interpreting the findings. Changes in the exposure variable over the follow-up period warrant further investigation. Fifth, our study focused solely on all-cause mortality and did not analyze cause-specific mortality. Future research should examine cause-specific mortality to better understand how social capital impacts different causes of death. Sixth, we used self-reported questionnaires to collect data, which may have introduced potential response biases in the data regarding subjective reports such as social capital and health conditions. In particular, there is an insufficient adjustment for the presence or severity of the disease. However, in the sensitivity analysis, the results did not change even when the Katz index, a basic ADL indicator, was added as a proxy variable for severe illness or disability. Finally, since this cohort study was conducted in only two regions of Myanmar, the generalizability of our findings is limited.

## Conclusions

In conclusion, our study identified a protective effect of social support on all-cause mortality among adults aged 60 and above in Myanmar. Specifically, receiving and providing instrumental support showed greater protection against mortality compared to emotional support, particularly in rural areas of Myanmar. These findings hold significant public health implications, suggesting that bolstering social support networks could be pivotal in enhancing survival among older adults. Therefore, integrating social support initiatives into health services for older adults in Myanmar could be a crucial strategy for improving survival rates.

## Supplementary Information

Additional file 1: Figure S1. Selection of the study participants for the Healthy and Active Ageing in Myanmar project. Recruitment process of participants from Yangon and Bago, resulting in 1,200 baseline participants in 2018. Figure S2. Hazard ratios of each variable on social capital for all-cause mortality. Hazard ratios for each variable of civic participation, social cohesion, and social support, in association with all-cause mortality. Figure S3. Hazard ratios of social capital for all-cause mortality stratified by residential area. Results of a stratified analysis, illustration the hazard ratios of social capital for all-cause mortality in Yangon and Bago.

Additional file 2: Table S1. Hazard ratios of social capital for all-cause mortality adjusted for all covariates plus basic ADL. Results of a sensitivity analysis, illustrating the association between social capital and all-cause mortality, with adjustments for all covariates and basic Activities of Daily Living (ADL).

## Data Availability

The dataset contains sensitive personal information capable of identifying individuals, thus classifying it as confidential. However, it may be shared solely within the context of collaborative research, provided that appropriate agreements and mutual understanding are established. This research is being conducted collaboratively by researchers from Japan and Myanmar, grounded in a mutual agreement. The data is owned by the designated representatives of both the Japanese and Myanmar research teams and is not intended for dissemination to third parties unless it serves to facilitate legitimate collaborative research efforts. The rationale for this restriction is that the data originates from a comprehensive epidemiological survey of the elderly population in Myanmar, encompassing information that can identify individuals. Even with the removal of personal identifiers, the current regulatory framework poses significant challenges to the release of the data.

## Abbreviations

JAGES: Japan Gerontological Evaluation Study
NCD: Non-communicable diseases
IADL: Instrumental activities of daily living
BMI: Body mass index
SRH: Self-rated health
SES: Socioeconomic status
ADL: Activities of daily living
HRs: Hazard ratios
95% CIs: 95% Confidence intervals
SD: Standard deviation
