# Supplementary material for: Association between social capital and mortality among community-dwelling older adults in Myanmar 2018–2022: a prospective cohort study
Source: BMC Glob Public Health. 2025 Mar 17;3:21. doi: 10.1186/s44263-025-00137-x (PMC11912608; doi:10.1186/s44263-025-00137-x)
Supplement: Supplementary file 1 — Additional file 1: Figure S1. Selection of the study participants for the Healthy and Active Ageing in Myanmar project. Recruitment process of participants from Yangon and Bago, resulting in 1,200 baseline participants in 2018. Figure S2. Hazard ratios of each variable on social capital for all-cause mortality. Hazard ratios for each variable of civic participation, social cohesion, and social support, in association with all-cause mortality. Figure S3. Hazard ratios of social capital for all-cause mortality stratified by residential area. Results of a stratified analysis, illustration the hazard ratios of social capital for all-cause mortality in Yangon and Bago. [file 44263_2025_137_MOESM1_ESM.pdf]

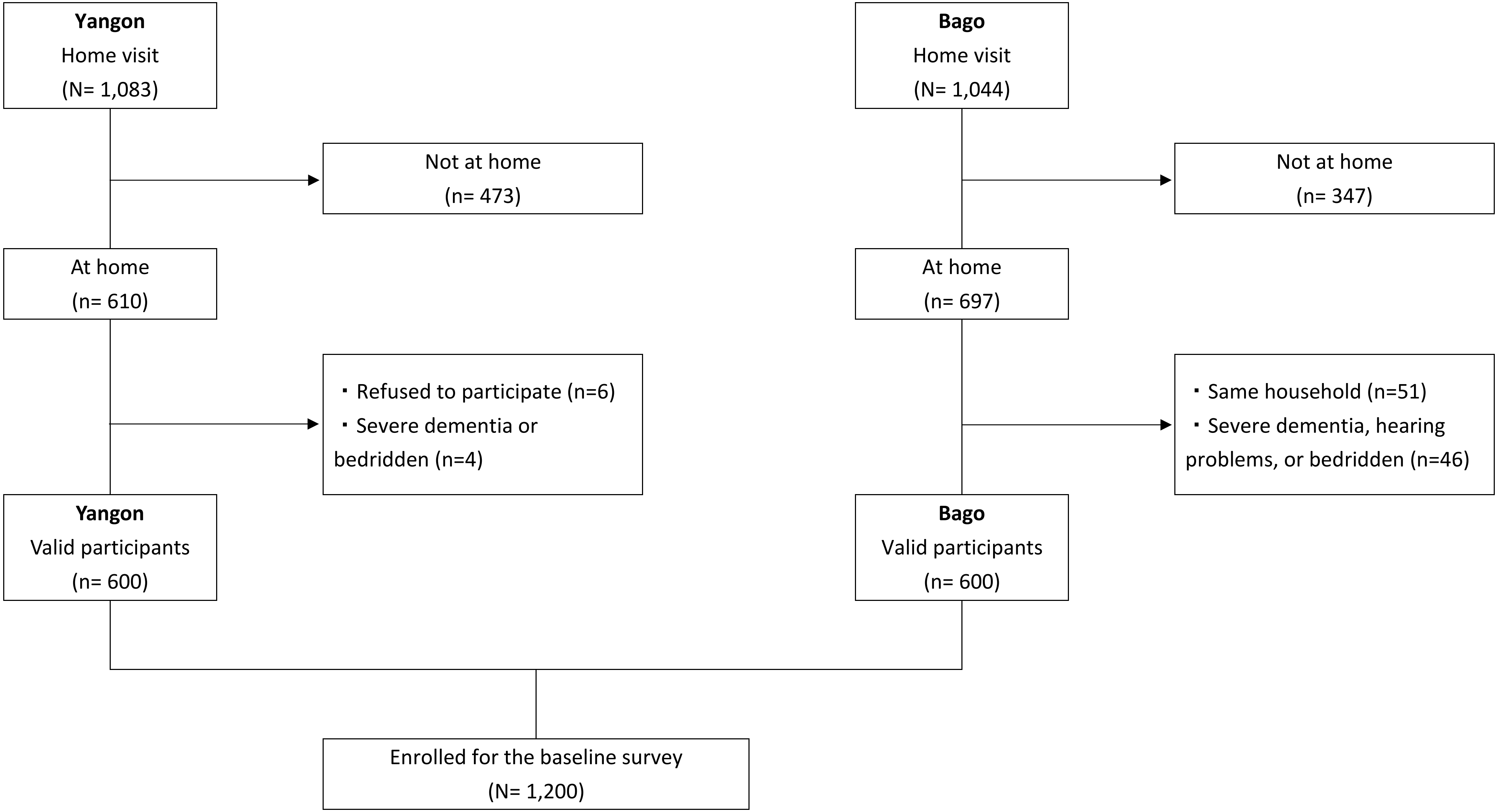

Figure S1. Selection of the study participants for the Healthy and Active Ageing in Myanmar project

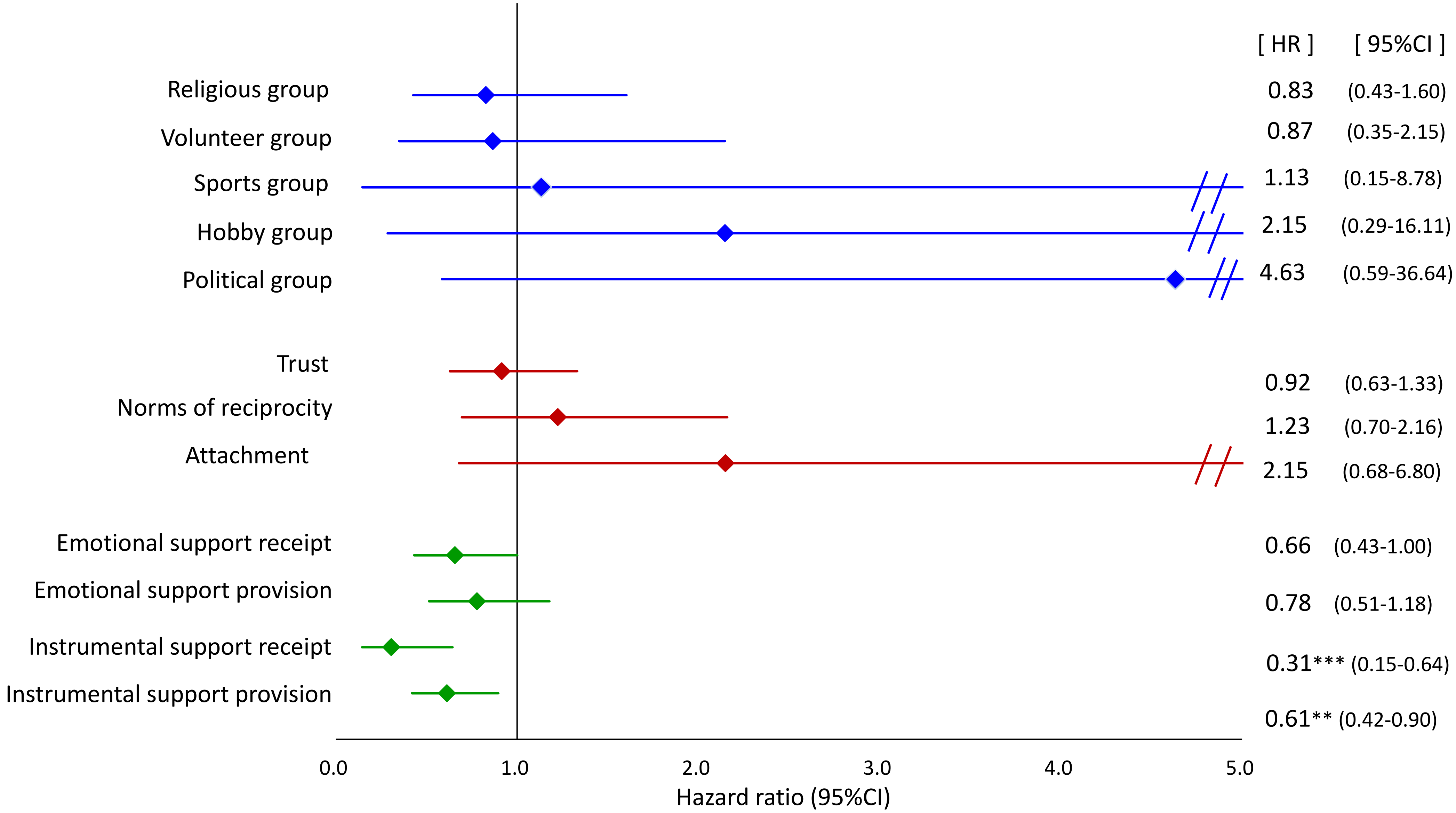

Figure S2. Hazard ratios of each variable on social capital for all-cause mortality

HR: Hazard ratio; CI: Confidence interval

*P<0.05; **P<0.01; ***P<0.001

Due to limited data on community meetings, survival analysis was inconclusive and could not be graphically represented. Multiple imputation by chained equations was performed using age, gender, education, wealth index, BMI, SRH, illness during preceding year, smoking history, alcohol intake, walking time, and residential area.

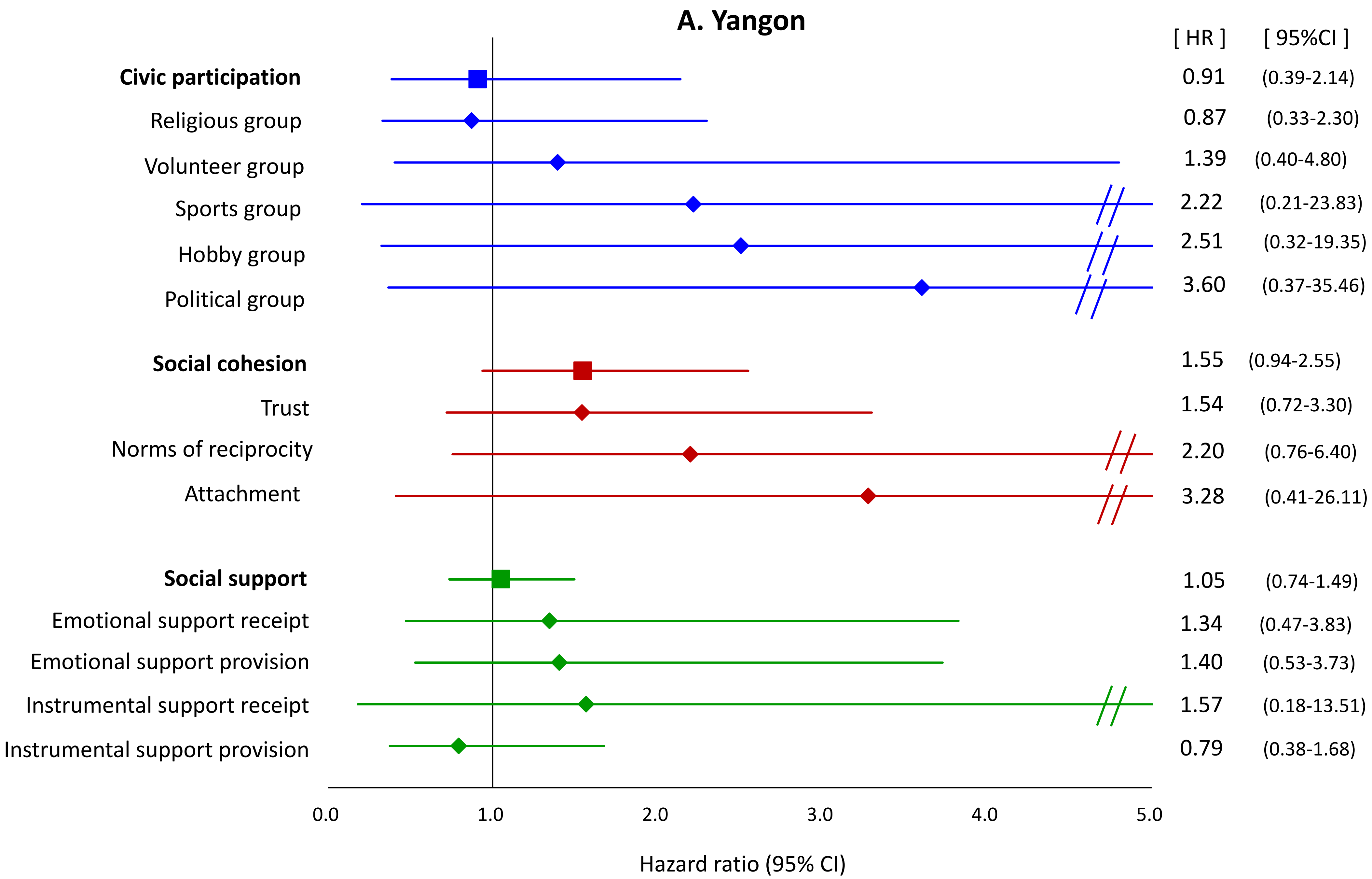

A. Yangon
[HR]
[95%CI]
Civic participation
0.91
(0.39-2.14)
Religious group
0.87
(0.33-2.30)
Volunteer group
1.39
(0.40-4.80)
Sports group
2.22
(0.21-23.83)
Hobby group
2.51
(0.32-19.35)
Political group
3.60
(0.37-35.46)
Social cohesion
1.55
(0.94-2.55)
Trust
1.54
(0.72-3.30)
Norms of reciprocity
2.20
(0.76-6.40)
Attachment
3.28
(0.41-26.11)
Social support
1.05
(0.74-1.49)
Emotional support receipt
1.34
(0.47-3.83)
Emotional support provision
1.40
(0.53-3.73)
Instrumental support receipt
1.57
(0.18-13.51)
Instrumental support provision
0.79
(0.38-1.68)
0.0
1.0
2.0
3.0
4.0
5.0
Hazard ratio (95% CI)

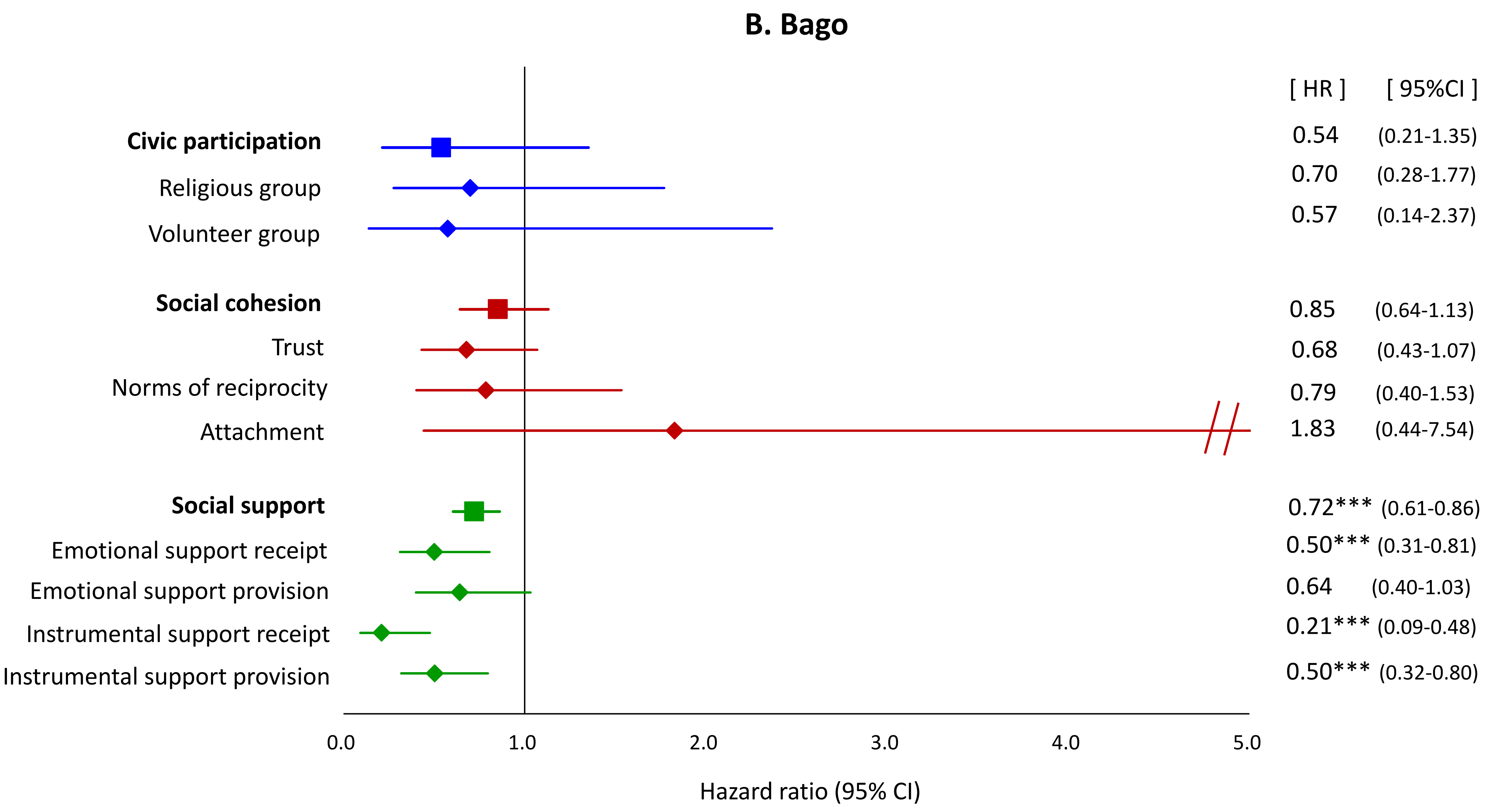

Figure S3. Hazard ratios of social capital for all-cause mortality stratified by residential area

Figure A: Hazard ratios of social capital for all-cause mortality in Yangon

Figure B: Hazard ratios of social capital for all-cause mortality in Bago

HR: Hazard ratio; CI: Confidence interval

*P<0.05; **P<0.01; ***P<0.001

Due to limited data on community meetings, survival analysis was inconclusive and could not be graphically represented. Multiple imputation by chained equations was performed using age, gender, education, wealth index, BMI, SRH, illness during preceding year, smoking history, alcohol intake, walking time, and residential area.
