# Supplementary material for: Association between social capital and mortality among community-dwelling older adults in Myanmar 2018–2022: a prospective cohort study
Source: BMC Glob Public Health. 2025 Mar 17;3:21. doi: 10.1186/s44263-025-00137-x (PMC11912608; doi:10.1186/s44263-025-00137-x)
Supplement: Supplementary file 2 — Additional file 2: Table S1. Hazard ratios of social capital for all-cause mortality adjusted for all covariates plus basic ADL. Results of a sensitivity analysis, illustrating the association between social capital and all-cause mortality, with adjustments for all covariates and basic Activities of Daily Living (ADL). [file 44263_2025_137_MOESM2_ESM.docx]

| **Table S1.** Hazard ratios of social capital for all-cause mortality adjusted for all covariates plus basic ADL | | | | | | | | | | | | | | | | | | | | | | | | | |
| --- | --- | --- | --- | --- | --- | --- | --- | --- | --- | --- | --- | --- | --- | --- | --- | --- | --- | --- | --- | --- | --- | --- | --- | --- | --- |
|  |  |  | |  | | |  | |  | | | |  | | |  | |  | | | |  | | |  |
|  |  | **Overall** | | | | | | **Yangon** | | | | | | | | | **Bago** | | | | | | | | |
|  |  | HR | 95%CI | | | | | HR | | | 95%CI | | | | | | HR | | | 95%CI | | | | | |
| Civic participation | No | 1.00 |  | |  | | | 1.00 | | |  | | |  | | | 1.00 | | |  | | |  | | |
|  | Yes | 0.70 | 0.37 | | 1.32 | | | 0.85 | | | 0.34 | | | 2.11 | | | 0.58 | | | 0.23 | | | 1.46 | | |
|  |  |  |  | |  | | |  | | |  | | |  | | |  | | |  | | |  | | |
| Social cohesion |  | 1.04 | 0.82 | | 1.33 | | | 1.47 | | | 0.87 | | | 2.48 | | | 0.91 | | | 0.69 | | | 1.21 | | |
|  |  |  |  | |  | | |  | | |  | | |  | | |  | | |  | | |  | | |
| Social support |  | 0.84* | 0.71 | | 0.98 | | | 1.03 | | | 0.72 | | | 1.49 | | | 0.78** | | | 0.64 | | | 0.94 | | |
| HR: Hazard ratio; CI: Confidence interval | | | | | | | | | | | | | | | | | | |  | |  | | |  | |
| **P*<0.05; ***P*<0.01 | | | | | |  | | | |  | |  | | |  | | | |  | |  | | |  | |
| Non-stratified model (Overall) was adjusted for age, gender, education, wealth index, BMI, SRH, illness during preceding year, smoking history, alcohol consumption, and walking time, residential area and Katz index. | | | | | | | | | | | | | | | | | | | | | | | | | |
| Stratified model (Yangon, Bago) was adjusted for age, gender, education, wealth index, BMI, SRH, illness during preceding year, smoking history, alcohol consumption, and walking time, and Katz index. | | | | | | | | | | | | | | | | | | | | | | | | | |
